# Trametinib as second-line therapy for advanced KRAS G12C-mutant non-small cell lung cancer: a single-center clinical analysis of 20 cases

**DOI:** 10.3389/fmed.2026.1851178

**Published:** 2026-05-28

**Authors:** Xinhui Wang, Junyan Yu

**Affiliations:** Heping Hospital Affiliated to Changzhi Medical College, Changzhi, China

**Keywords:** KRAS G12C mutation, MEK inhibitor, non-small cell lung cancer, second-line therapy, trametinib

## Abstract

**Objective:**

To investigate the clinical efficacy and safety of the MEK1/2 inhibitor trametinib in the second-line treatment of advanced non-small cell lung cancer (NSCLC) with a KRAS G12C mutation and to explore potential clinical factors associated with treatment response.

**Methods:**

A retrospective analysis was performed on 20 patients with advanced NSCLC harboring the KRAS G12C mutation who were admitted to Heping Hospital, which is affiliated with Changzhi Medical College, from January 2020 to June 2023. After disease progression following first-line platinum-based doublet chemotherapy combined with PD-1/PD-L1 inhibitor therapy, all patients received 2 mg of trametinib orally once daily, with 21 days defined as one treatment cycle, until disease progression, death, or intolerable toxicity occurred. The primary endpoint was the objective response rate (ORR), and the secondary endpoints included the disease control rate (DCR), progression-free survival (PFS), overall survival (OS), and treatment-related adverse reactions.

**Results:**

Among the 20 patients, 16 were male, and 4 were female, with a median age of 65 years (range 51–78 years); 18 patients had a history of smoking, and the predominant histological type was adenocarcinoma (19 cases). The ORR was 27.8% (5/18), and the DCR was 72.2% (13/18). The median follow-up duration was 10.2 months, the median PFS was 3.8 months (95% CI: 2.9–4.7 months), and the median OS was 8.6 months (95% CI: 6.4–10.8 months). Univariate analysis indicated that the ORR of patients without bone metastasis was significantly greater than that of patients with bone metastasis (35.7% vs. 0%, *P* = 0.042). The DCR was greater in patients with PD-L1 expression ≥1% (81.8% vs. 50.0%, *P* = 0.039). The most common treatment-related adverse reactions in the overall cohort were rash (35.0%), diarrhea (25.0%), and fatigue (20.0%). The incidence of grade 3 adverse reactions was 15.0%. No grade 4 or higher adverse reactions or treatment-related deaths were observed.

**Conclusion:**

Trametinib, as a second-line treatment for advanced NSCLC with a KRAS G12C mutation, has demonstrated evident anti-tumor activity, manageable toxicity, and favorable tolerability; exploratory analysis indicated that the absence of bone metastasis and PD-L1 positivity might be associated with improved outcomes. This regimen is highly clinically accessible and may serve as a second-line treatment option when KRAS G12C-specific inhibitors are unavailable. Its clinical value requires further validation through large-sample prospective studies.

## 1 Introduction

Lung cancer continues to be the leading malignant disease worldwide in terms of both incidence and mortality. Among the pathological categories of lung cancer, non-small cell lung cancer (NSCLC) constitutes the majority of cases, accounting for about 80%–85% of all lung cancers. Clinical evidence indicates that nearly 70% of patients with NSCLC are initially diagnosed at a locally advanced stage or with distant metastases; thus, the opportunity for curative surgical intervention is missing. As a result, comprehensive treatment approaches based on systemic therapy have become the main strategy for improving patient prognosis (1). The RAS gene family (rat sarcoma viral oncogene homolog) represents the most frequently altered oncogenic driver family in human cancers. Among these genes, KRAS (Kirsten rat sarcoma viral oncogene) has received substantial clinical attention because of its high mutation frequency (2). In NSCLC, mutations at codon 12 of the KRAS gene (including subtypes such as G12C, G12D, and G12V) are observed in more than 30% of advanced lung adenocarcinoma cases. Numerous investigations have demonstrated that these mutation subtypes are strongly associated with a smoking history, elevated tumor mutational burden, and an immunosuppressive tumor microenvironment, ultimately resulting in poor prognosis and reduced therapeutic response rates (3). Among the different KRAS codon 12 mutation subtypes, the KRAS G12C mutation has become the first successfully targeted subtype because its molecular configuration contains a Switch-II binding pocket that enables covalent interaction. Currently, specific covalent inhibitors such as sotorasib and adagrasib have been approved for clinical application and have significantly improved survival outcomes in this patient population. However, in real-world clinical settings, multiple factors–including limited drug availability, high financial burden, primary or acquired resistance, and treatment contraindications–restrict many patients from receiving KRAS G12C inhibitors. Therefore, alternative therapeutic strategies that are effective, less toxic, and more accessible are critically needed (4). KRAS mutations can aberrantly activate the RAS-RAF-MEK-ERK signaling cascade (mitogen-activated protein kinase pathway), continuously promoting tumor cell proliferation, invasion, and resistance to apoptosis. Within this cascade, MEK functions as a key node that links RAF and ERK. Trametinib is a highly selective oral allosteric inhibitor of MEK1/2 that can effectively block MEK kinase activity and suppress downstream ERK phosphorylation, thereby interrupting abnormal pathway activation driven by KRAS mutations. It has already been approved for clinical use in multiple malignancies, including melanoma and NSCLC (5, 6). Nevertheless, evidence concerning trametinib monotherapy as a second-line treatment for KRAS G12C-mutant NSCLC in Chinese populations remains limited, particularly real-world data from primary medical institutions concerning its efficacy, safety, and predictive biomarkers of response. Therefore, in this study, a single-center retrospective analysis was performed to evaluate the efficacy and safety of trametinib monotherapy as second-line therapy in patients with advanced KRAS G12C-mutant NSCLC for whom first-line platinum-based chemotherapy combined with immunotherapy failed. In addition, potential clinical factors that may be related to treatment response were explored to provide real evidence to support the clinical decision-making of this population.

## 2 Materials and methods

### 2.1 General information

**FIGURE 1:**
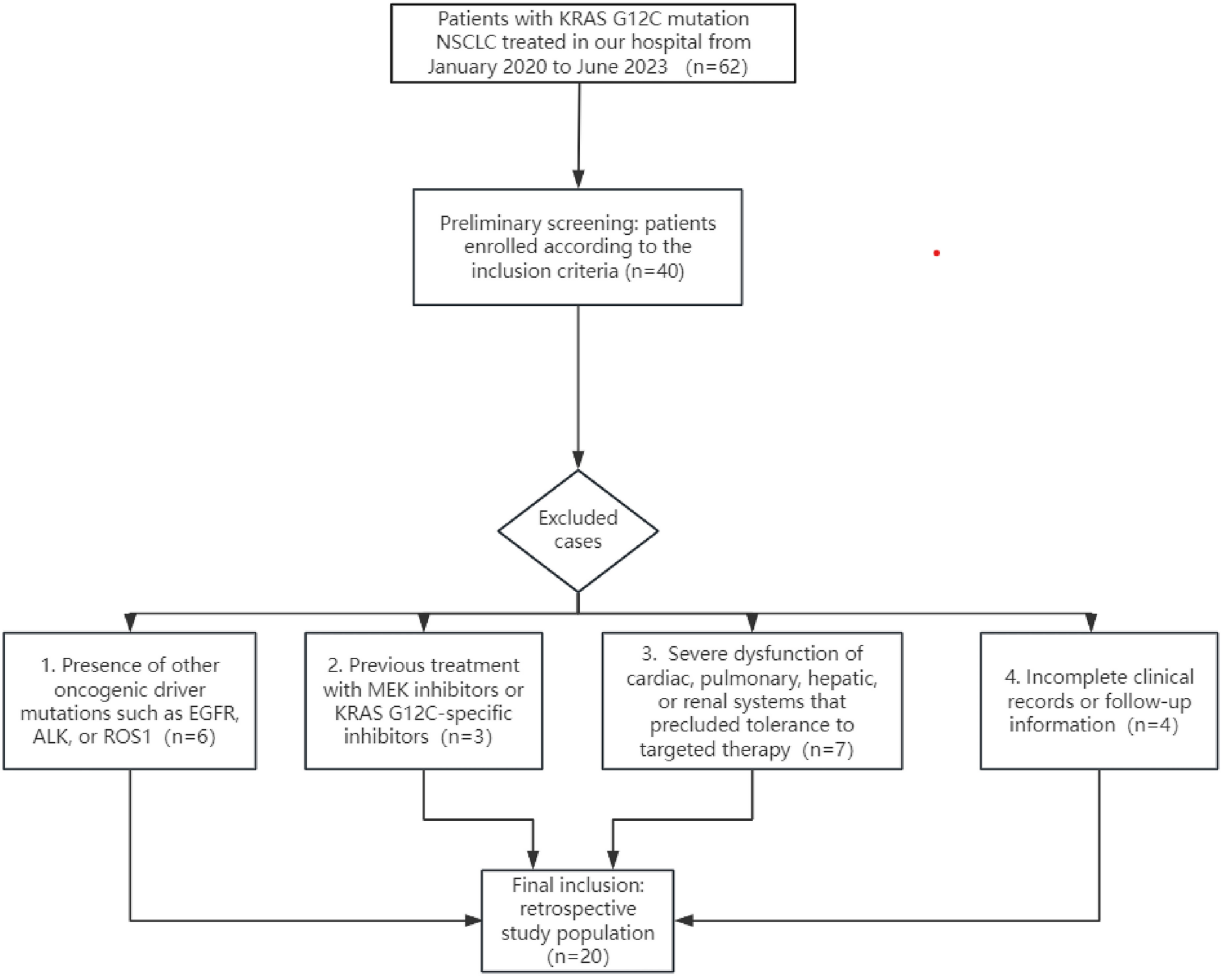
Trial profile.

A total of 20 patients with advanced KRAS G12C-mutant NSCLC treated at the Department of Medical Oncology at Heping Hospital Affiliated with Changzhi Medical College between January 2020 and June 2023 were retrospectively enrolled. The inclusion criteria were as follows: ➀ histologically or cytologically confirmed NSCLC, classified as stage IIIB–IV according to the AJCC 8th Edition TNM Staging System for Non-Small Cell Lung Cancer; ➁ the presence of a KRAS G12C mutation confirmed by tissue or blood-based testing; ➂ disease progression or intolerance following first-line platinum-based doublet chemotherapy combined with PD-1/PD-L1 inhibitors; ➃ an Eastern Cooperative Oncology Group (ECOG) performance status (PS) score of 0–2; and ➄ at least one measurable target lesion defined according to the RECIST 1.1 criteria. The exclusion criteria were as follows: ➀ the presence of other oncogenic driver mutations, such as those in EGFR, ALK, or ROS1; ➁ previous treatment with MEK inhibitors or KRAS G12C-specific inhibitors; ➂ severe dysfunction of the cardiac, pulmonary, hepatic, or renal system that precluded tolerance to targeted therapy; and ➃ incomplete clinical records or follow-up information. The patient selection process is shown in Figure 1. This study was approved by the ethics committee of Peace Hospital Affiliated with Changzhi Medical College (approval number: [(2026)090]) and strictly abided by the ethical guidelines of the Declaration of Helsinki. In view of the retrospective design of this study, all the patient data were clinical data after anonymous processing, and no intervention beyond conventional diagnosis and treatment was involved. The ethics committee approved the exemption of patients’ informed consent.

### 2.2 Treatment regimen

All patients received trametinib as monotherapy. Trametinib (manufactured by GlaxoSmithKline, 2 mg/tablet) was administered orally at a dose of 2 mg once daily, either under fasting conditions or after meals. A treatment cycle was defined as 21 days. Therapy was continued until disease progression, death, or the development of intolerable toxicity. The dose adjustment principles were as follows: When grade 2 treatment-related adverse events occurred, treatment was temporarily suspended. After the adverse events recovered to grades 0–1, the dose was reduced to 1.5 mg orally once daily. When grade 3 or higher treatment-related adverse events occurred, trametinib was permanently discontinued.

### 2.3 Efficacy and safety evaluation

#### 2.3.1 Efficacy evaluation

Radiological assessments, including chest CT, were conducted every two treatment cycles (6 weeks) to evaluate treatment response based on the RECIST 1.1 criteria. Treatment responses were classified as complete response (CR), partial response (PR), stable disease (SD), or progressive disease (PD). Primary endpoint: The objective response rate (ORR) was defined as the proportion of evaluable patients who achieved CR or PR. Secondary endpoints included the disease control rate (DCR), defined as the proportion of evaluable patients who achieved CR, PR, or SD; progression-free survival (PFS), defined as the time from the initiation of trametinib therapy to the first occurrence of disease progression or death; and overall survival (OS), defined as the time from the initiation of trametinib therapy to death.

#### 2.3.2 Safety evaluation

Patients were monitored during each treatment cycle through outpatient follow-up or hospitalization to document treatment-related adverse events. The severity of adverse events was graded according to version 5.0 of the Common Terminology Criteria for Adverse Events (CTCAE 5.0) issued by the National Cancer Institute. The type, incidence, severity, and dose modifications associated with adverse events were recorded.

### 2.4 Statistical methods

Statistical analyses were conducted using SPSS version 27.0. Categorical variables are presented as frequencies and percentages (n, %), and comparisons between groups were performed using Fisher’s exact test. Survival data were analyzed using the Kaplan–Meier method, and corresponding survival curves were generated. Differences between survival curves were assessed using the log-rank test. A *P*-value < 0.05 was considered to indicate statistical significance. Owing to the small sample size and the limited number of events, the univariate analysis of all potential predictors was exploratory, and multivariate analysis was not conducted.

## 3 Results

### 3.1 Baseline characteristics of patients

A total of 20 patients with advanced KRAS G12C-mutant non-small cell lung cancer who fulfilled the inclusion and exclusion criteria were included in this study. The baseline clinicopathological characteristics of these patients are summarized in Table 1.

**TABLE 1:** Baseline characteristics of 20 patients with KRAS G12C-mutant advanced NSCLC.

Characteristics	*n* = 20	Percentage (%)	Characteristics	*n* = 20	Percentage (%)
Sex			Metastatic sites*		
Male	16	80	Bone	8	40
Female	4	20	Brain	3	15
Age (years)			Liver	2	10
<65	9	45	Adrenal gland	1	5
≥65	11	55	Pleura	6	30
Median age (range)	65 (51–78)	–	PD-L1 expression (*n* = 18)		
Smoking history			<1%	7	38.9
Never smoked	2	10	1%–49%	6	33.3
Former/current smoker	18	90	≥50%	5	27.8
Histological type			Co-mutations (*n* = 18)		
Adenocarcinoma	19	95	TP53	12	66.7
Squamous carcinoma	1	5	STK11	5	27.8
Clinical stage			KEAP1	4	22.2
Stage IIIB	3	15	Previous first-line regimen		
Stage IV	17	85	Platinum + PD-1 inhibitor	18	90
ECOG score			Platinum + PD-L1 inhibitor	2	10
0	6	30			
1	12	60			
2	2	10			

*Single patient may have multiple metastatic sites. ECOG: ECOG Performance Status. Clinical staging was based on the AJCC 8th Edition TNM Staging System for Non-Small Cell Lung Cancer.

### 3.2 Short-term efficacy

Among the 20 patients, 2 patients were excluded from the efficacy evaluation set because of insufficient follow-up duration. Finally, 18 patients were evaluated for treatment response. As of the last follow-up in December 2023, the median follow-up duration for the entire cohort was 10.2 months. Among the 18 evaluated patients, no cases of CR were observed. Five patients achieved a PR, eight patients achieved SD, and five patients exhibited PD. The ORR of the entire cohort was 27.8% (5/18), and the DCR was 72.2% (13/18).

### 3.3 Survival outcomes

**FIGURE 2:**
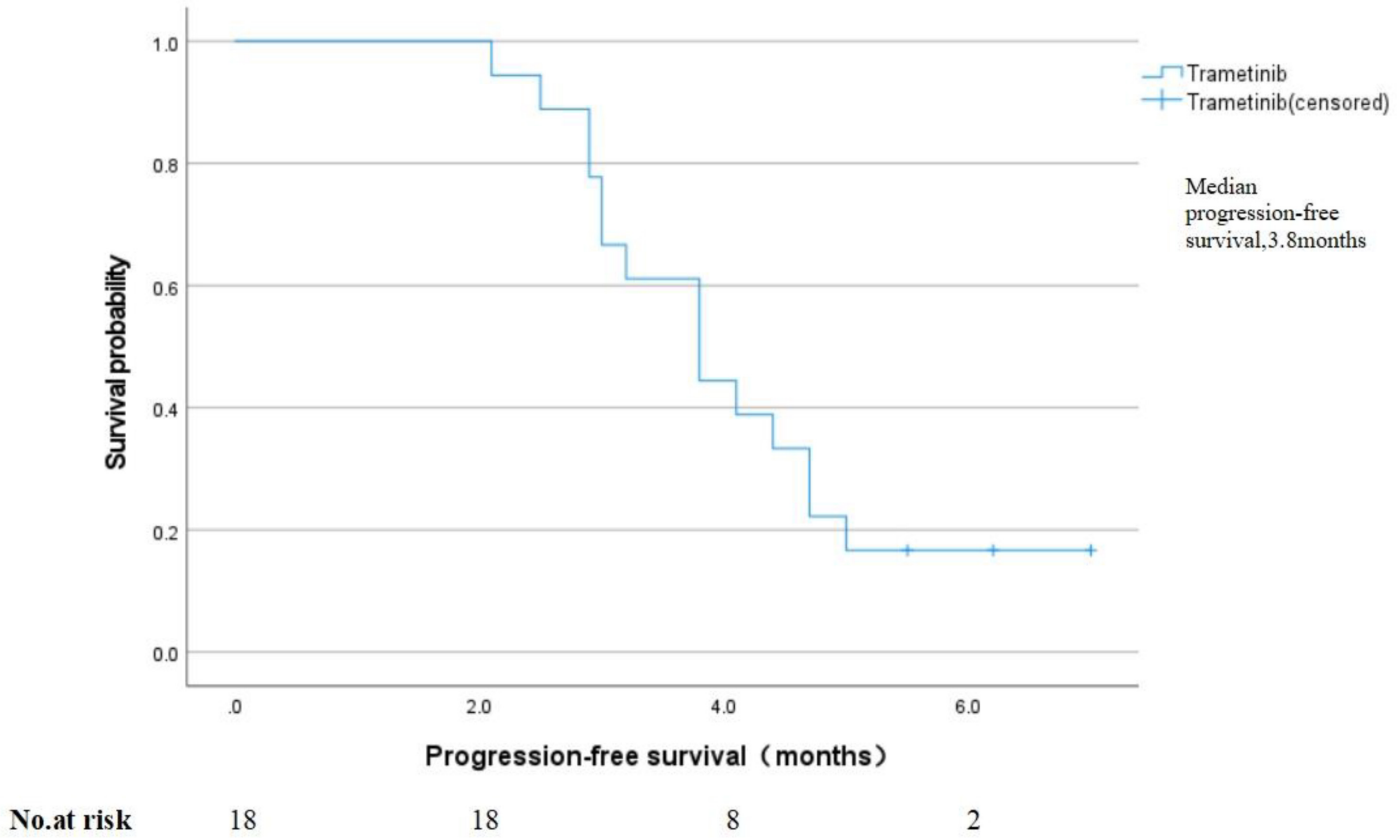
Kaplan–Meier curve for progression-free survival.

**FIGURE 3:**
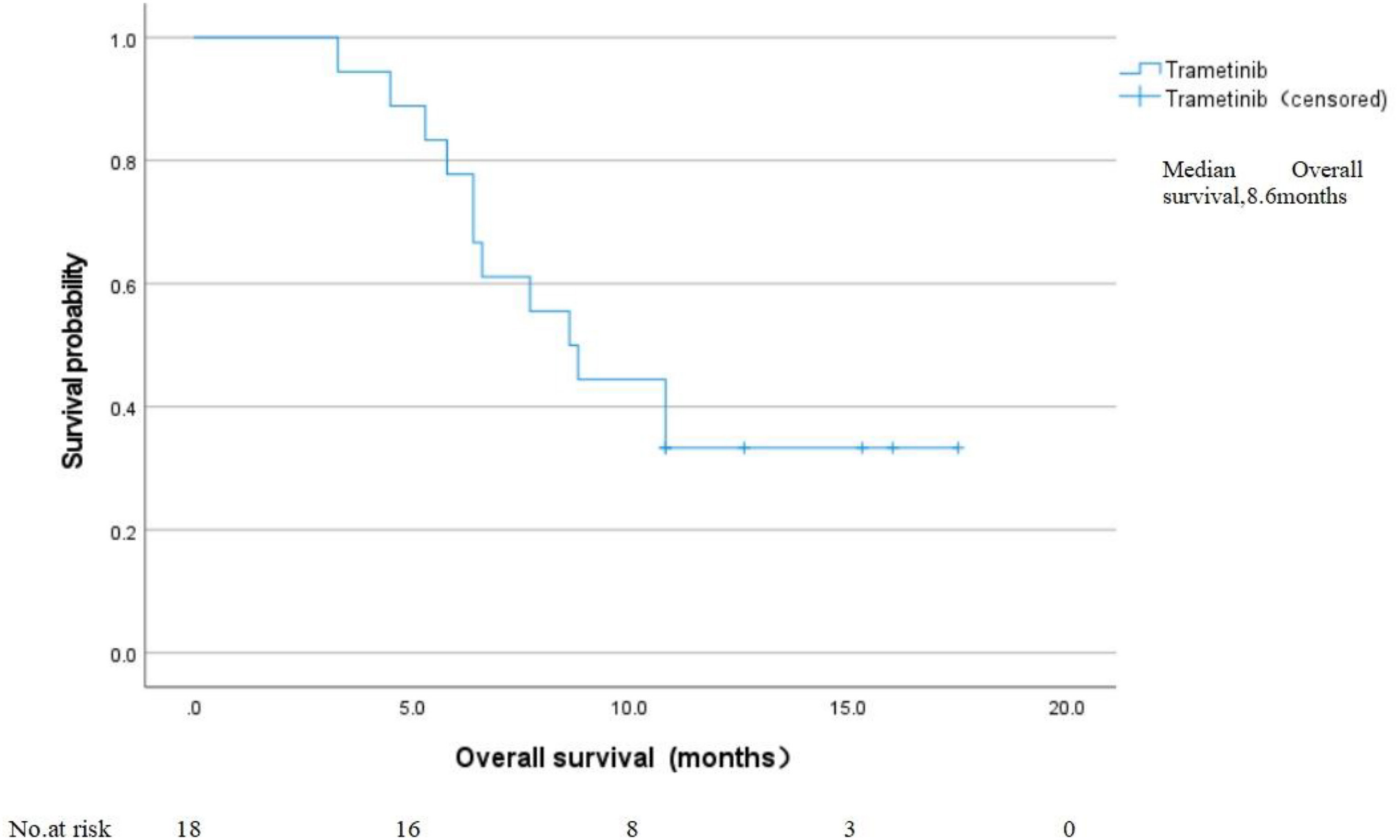
Kaplan–Meier curve for overall survival.

Among the 18 evaluable patients, 15 patients reached the PFS endpoint, and 12 patients reached the OS endpoint. Kaplan–Meier survival analysis revealed that the median PFS was 3.8 months (95% CI: 2.9–4.7 months), and the median OS was 8.6 months (95% CI: 6.4–10.8 months). The results are presented in Figures 2, 3.

### 3.4 Univariate exploratory analysis of clinical factors associated with treatment response

Potential factors affecting treatment efficacy, including sex, age, smoking history, the presence of bone metastasis, the presence of brain metastasis, PD-L1 expression, and co-mutations of TP53, STK11, and KEAP1, were included in the univariate exploratory analysis. The results indicated that patients without bone metastasis had a significantly higher ORR than those with bone metastasis did (35.7% vs. 0%, *P* = 0.042). In addition, patients with PD-L1 expression ≥1% had a significantly greater DCR than those with PD-L1 expression <1% (81.8% vs. 50.0%, *P* = 0.039). However, age, sex, smoking history, brain metastasis status, and co-mutation status of TP53/STK11/KEAP1 were not significantly associated with the ORR or DCR in univariate comparisons (all *P* > 0.05). However, given the small number of events and the unadjusted nature of the analysis, these findings should not be interpreted as definitive predictive biomarkers. The results are presented in Table 2.

**TABLE 2:** Univariate analysis of factors influencing the efficacy of trametinib.

Variable	Evaluable cases	ORR (%)	DCR (%)	*P*-value (ORR)	*P*-value (DCR)
Sex					
Male	16	25.0	68.8	0.680	0.550
Female	2	50.0	100.0		
Age					
<65 years	9	33.3	77.8	0.510	0.720
≥65 years	9	22.2	66.7		
Smoking history					
Yes	16	25.0	68.8	0.680	0.550
No	2	50.0	100.0		
Bone metastasis					
Yes	6	0	50.0	0.042*	0.208
No	12	35.7	83.3		
Brain metastasis					
Yes	3	0	66.7	0.530	0.910
No	15	33.3	73.3		
PD-L1 expression					
<1%	6	16.7	50.0	0.390	0.039*
≥1%	11	36.4	81.8		
TP53 mutation					
Yes	10	30.0	70.0	0.750	0.680
No	8	25.0	75.0		
STK11 mutation					
Yes	5	20.0	60.0	0.680	0.550
No	13	30.8	76.9		
KEAP1 mutation					
Yes	4	25.0	50.0	0.910	0.280
No	14	28.6	78.6		

*P < 0.05 indicates a statistically significant difference.

### 3.5 Safety analysis

All 20 patients were included in the safety analysis. The most common treatment-related adverse events were rash (35.0%), diarrhea (25.0%), and fatigue (20.0%). Grade 3 treatment-related adverse events occurred in three patients (15.0%), including rash in two patients and diarrhea in one patient. No grade 4 or higher treatment-related adverse events or treatment-related deaths were observed. Dose adjustments due to adverse events were required in two patients, and permanent treatment discontinuation occurred in one patient. All adverse events improved following symptomatic and supportive treatment, and no persistent severe adverse events were observed. Overall, treatment-related adverse events were predominantly grade 1–2, and the regimen was generally well tolerated. The detailed results are presented in Table 3.

**TABLE 3:** Treatment-related adverse events in 20 patients.

Adverse event	Grade 1–2 cases (%)	Grade 3 cases (%)	Total incidence (%)
Rash	5 (25.0)	2 (10.0)	35.0
Diarrhea	4 (20.0)	1 (5.0)	25.0
Fatigue	4 (20.0)	0 (0)	20.0
Nausea/vomiting	2 (10.0)	0 (0)	10.0
Stomatitis	1 (5.0)	0 (0)	5.0
Peripheral edema	1 (5.0)	0 (0)	5.0

Adverse events were graded according to CTCAE version 5.0. No grade 4 or 5 adverse events were observed.

## 4 Discussion

KRAS mutations were historically regarded as “undruggable” targets in the context of targeted therapy for non-small cell lung cancer. This therapeutic limitation was not overcome until the successful development of covalent inhibitors specifically targeting the KRAS G12C mutation. At present, agents such as sotorasib and adagrasib have been approved for use in later-line treatment of advanced KRAS G12C-mutant NSCLC. Data from their pivotal clinical trials indicate that the ORR of single-agent therapy in the later-line setting ranges from 37.1% to 42.9%, with a median PFS of about 6.5–6.8 months, which is markedly superior to that of conventional chemotherapy (7). However, in real-world clinical practice in China, these agents continue to face several practical challenges, including uneven drug accessibility across regions, the emergence of drug resistance, and a substantial financial burden for patients. Therefore, alternative therapeutic approaches are urgently needed. This study primarily evaluated the clinical efficacy of the MEK1/2 inhibitor trametinib as a second-line monotherapy in patients with advanced KRAS G12C-mutant NSCLC. By incorporating an analysis of predictive biomarkers associated with treatment response, this study aims to provide a more feasible and accessible therapeutic option for this patient population.

### 4.1 Sensitivity of the KRAS G12C mutation subtype to MEK inhibitors

The findings of this study demonstrated that second-line trametinib monotherapy achieved an ORR of 27.8% and a DCR of 72.2% in patients with KRAS G12C-mutant NSCLC, with a median PFS of 3.8 months and a median OS of 8.6 months. These therapeutic outcomes were clearly superior to those reported in the international multicenter phase II trial by Blumenschein et al. (8), in which the overall KRAS-mutant cohort achieved an ORR of 12% and a median PFS of 3.7 months. These observations suggest that the KRAS G12C mutation subtype may exhibit enhanced sensitivity to inhibition of the MEK signaling pathway. These conclusions are consistent with those of preclinical studies indicating that different KRAS mutation subtypes display variable sensitivity to MEK inhibition. The underlying mechanism may be associated with the structural conformation of the G12C mutant protein and its distinct dependence on downstream signaling pathways (9). However, in the current NCCN guidelines, the first-line treatment for advanced NSCLC with a KRAS G12C mutation is based on immune checkpoint inhibitors, and approved sotorasib and adagrasib are recommended only as back-line treatments. The results of phase II studies revealed that ORR was 37.1% and 42.9%, and the median OS was 12.5 months and 12.6 months, respectively (10–12). The randomized phase III codebreak 200 trial further confirmed the superiority of sotorasibu’s PFS over docetaxel (5.6 months vs. 4.5 months, HR = 0.66) (13). Notably, the efficacy of the specific KRAS G12C inhibitor, namely, trimetinib, in this study is lower than that of the abovementioned KRAS G12C inhibitor, but the above comparison is an indirect comparison across studies. Owing to the single-arm and retrospective design of this study, it is not possible to infer which is better. However, this difference in efficacy cannot negate the clinical necessity of this study. In the real world, KRAS G12C inhibitors are not associated with medical insurance, high cost, unbalanced regional supply or inevitable acquired drug resistance. As a result, many patients face a “treatment vacuum” after disease progression, and there is an urgent need for accessible and effective alternatives to avoid relying solely on the best supportive treatment. As an oral MEK inhibitor that has been included in the national medical insurance catalog of China, the average monthly treatment cost of trimetinib is only a few times that of imported KRAS G12C-targeted drugs, which can be routinely prescribed and monitored in primary hospitals; moreover, the incidence of grade 3 adverse events in this study was only 15.0%, and there was no treatment-related death. The toxicity spectrum revealed mainly easy-to-handle reactions such as rash and diarrhea. This low toxicity, safety and convenient management feature has the advantages of palliative treatment and quality of life that cannot be ignored for people whose physical condition has decreased or whose accumulated toxicity is obvious after first-line immunochemotherapy. In summary, the goal of this study is not to challenge the status of the existing standard back-line treatment but to systematically report the real efficacy and safety baseline of trimetazidine as a second-line MEK inhibitor for the first time when a KRAS G12C inhibitor is not timely, which fills the evidence-based gap in areas with limited medical resources and provides key clinical assumptions and a feasible basis for follow-up prospective head-to-head studies such as those involving trimetazidine combined with immunotherapy or local radiotherapy.

### 4.2 Exploration of predictive biomarkers for treatment efficacy

In the present study, exploratory univariate analysis suggested two potential factors associated with treatment response, namely, bone metastasis status and PD-L1 expression level. The results indicated that patients with bone metastasis exhibited an ORR of 0% following trametinib monotherapy, which was significantly lower than that observed in patients without bone metastasis, suggesting that bone metastasis may act as a negative predictive factor for trametinib efficacy. The possible mechanisms underlying this phenomenon may involve two aspects. First, the penetration of trametinib into bone tissue may be relatively limited, resulting in insufficient drug concentrations within bone metastatic lesions (14). Second, the bone marrow microenvironment in patients with bone metastases is characterized by strong immunosuppressive properties, which may further weaken the anti-tumor effects of MEK inhibitors (15). These findings imply that in patients with KRAS G12C-mutant NSCLC accompanied by bone metastases, the clinical benefit of trametinib monotherapy may be restricted. In clinical settings, combination treatment strategies, including local radiotherapy, bone-targeting agents, or other systemic therapies, may be considered to enhance overall therapeutic efficacy. In addition, this study demonstrated that patients with PD-L1 expression ≥1% had a significantly greater DCR than those with PD-L1-negative tumors, suggesting that PD-L1 expression may serve as a predictive biomarker for trametinib efficacy. Previous preclinical investigations have shown that MEK inhibitors can inhibit the MAPK signaling pathway, thereby increasing the expression of MHC-I molecules on tumor cell surfaces and restoring antigen presentation capacity. Moreover, they may reduce the infiltration of immunosuppressive cells within the tumor microenvironment, thereby improving immune conditions and producing synergistic anti-tumor effects when combined with immune checkpoint inhibitors (16). These findings provide a theoretical rationale for future studies evaluating trametinib combined with immunotherapy in patients with PD-L1–positive KRAS G12C-mutant NSCLC, which may result in improved clinical outcomes. Furthermore, in this study, no significant association was detected between common co-mutations–such as those in TP53, STK11, and KEAP1–and the therapeutic response to trametinib. These results are inconsistent with findings from the SWOG S1507 study, in which TP53 mutation was associated with poorer prognosis (17). This discrepancy may stem from the small sample size and short follow-up period, necessitating further verification in larger cohorts. Given the retrospective design, restricted sample volume and absence of multivariate adjustment, these biomarker results are only hypothesis-driven and cannot serve as definitive evidence for clinical practice.

### 4.3 Safety and clinical accessibility analysis

In this study, the overall safety profile of trametinib was favorable. The most frequently observed adverse events were grade 1–2 rash, diarrhea, and fatigue. The incidence of grade 3 adverse events was only 15.0%, and no grade 4 or higher adverse events or treatment-related deaths were reported. Only one patient permanently discontinued treatment because of adverse events. These findings are largely consistent with the safety outcomes reported in previous registration trials of trametinib across various tumor types (17, 18). The adverse-event profile of this regimen is relatively clear and manageable in routine clinical practice. In addition, it does not require complex laboratory monitoring, allowing safe administration even in primary healthcare settings and facilitating broader clinical application. From a pharmacoeconomic perspective, trametinib has been included in the National Reimbursement Drug List (NRDL, Category B) in China. Following reimbursement, the monthly out-of-pocket cost for patients is significantly lower than that associated with imported KRAS G12C-targeted inhibitors, thereby reducing the economic burden. For patients with KRAS G12C-mutant NSCLC in primary healthcare settings or those with financial constraints, trametinib provides advantages in terms of efficacy, safety, and cost-effectiveness, thus helping to address unmet clinical demands in this population.

### 4.4 Study limitations and future perspectives

This study has several limitations. First, the sample size is small (*n* = 20), and it is a retrospective and single-center study, which limits the reliability of the statistical analysis, selection bias, and the external validity of the research results and exploratory biomarker evaluation. Second, owing to the lack of a parallel control group, it is difficult to directly compare the efficacy of trimetinib and standard second-line therapy. Third, owing to the limited number of events and the lack of multivariate analysis, evaluating the independent predictive value of any clinical factors is impossible. Fourth, the lack of dynamic ctDNA monitoring limits the understanding of the mechanism of drug resistance. Therefore, the comparative advantages of its efficacy and safety need to be further studied. On the basis of the current findings, future multicenter, prospective, large-scale clinical studies are needed to more comprehensively evaluate the clinical value of trametinib in patients with advanced KRAS G12C-mutant NSCLC. In addition, further investigations of combination strategies–such as trametinib combined with immunotherapy, chemotherapy, or KRAS G12C inhibitors–may help overcome the limitations associated with monotherapy, particularly in patients with PD-L1-positive tumors or bone metastases. These strategies may ultimately provide more effective personalized treatment options for patients with KRAS G12C-mutant NSCLC.

## 5 Conclusion

In this small retrospective study, second-line trametinib monotherapy clearly exhibited anti-tumor activity in patients with advanced KRAS G12C-mutant NSCLC for whom first-line platinum-based chemotherapy combined with immunotherapy failed. The treatment-related adverse events were controllable, and the overall tolerance was acceptable. Preliminary observations revealed that patients without bone metastasis and with a PD-L1 expression ≥1% had a greater benefit trend, which needs to be verified prospectively. The scheme has advantages in terms of drug accessibility and simplified clinical management. When a specific KRAS G12C inhibitor is unavailable, it can be used as an alternative to second-line treatment. However, its clinical value still needs to be further confirmed by large-scale prospective studies.

## Data Availability

The original contributions presented in this study are included in this article/supplementary material, further inquiries can be directed to the corresponding author.

## References

[1] Bray F Laversanne M Sung H Ferlay J Siegel RL Soerjomataram I et al. Global cancer statistics 2022: GLOBOCAN estimates of incidence and mortality worldwide for 36 cancers in 185 countries. *CA Cancer J Clin.* (2024) 74:229–63. 10.3322/caac.21834 38572751

[2] Awad MM Liu S Rybkin II Arbour KC Julien Dilly MS Zhu VW et al. Acquired Resistance to KRAS(G12C) Inhibition in Cancer. *N Engl J Med.* (2021) 384:2382–93. 10.1056/NEJMoa2105281 34161704 PMC8864540

[3] Liu C Zheng S Wang Z Wang S Wang X Yang L et al. KRAS-G12D mutation drives immune suppression and the primary resistance of anti-PD-1/PD-L1 immunotherapy in non-small cell lung cancer. *Cancer Commun.* (2022) 42:828–47. 10.1002/cac2.12327 35811500 PMC9456691

[4] Torres-Jiménez J Espinar JB De Cabo HB Berjaga MZ Esteban-Villarrubia J Fraile JZ et al. Targeting KRAS(G12C) in non-small-cell lung cancer: current standards and developments. *Drugs.* (2024) 84:527–48. 10.1007/s40265-024-02030-7 38625662

[5] Planchard D Besse B Groen HJM Hashemi SMS Mazieres J Kim TM et al. Phase 2 study of dabrafenib plus trametinib in patients with BRAF V600E-Mutant metastatic NSCLC: updated 5-Year survival rates and genomic analysis. *J Thorac Oncol.* (2022) 17:103–15. 10.1016/j.jtho.2021.08.011 34455067

[6] Long GV Hauschild A Santinami M Kirkwood JM Atkinson V Mandala M et al. Final results for adjuvant dabrafenib plus trametinib in stage III melanoma. *N Engl J Med.* (2024) 391:1709–20. 10.1056/NEJMoa2404139 38899716

[7] Cantor DJ Aggarwal C. Targeting KRAS-Mutated NSCLC: novel TKIs and beyond. *Clin Cancer Res.* (2023) 29:3563–5. 10.1158/1078-0432.CCR-23-1658 37466928 PMC10530517

[8] Blumenschein GR Jr. Smit EF Planchard D Kim DW Cadranel J De Pas T et al. A randomized phase Ii study of the MEK1/MEK2 inhibitor trametinib (GSK1120212) compared with docetaxel in KRAS-mutant advanced non-small-cell lung cancer (NSCLC)†. *Ann Oncol.* (2015) 26:894–901. 10.1093/annonc/mdv072 25722381 PMC4855243

[9] Misale S Fatherree JP Cortez E Li C Bilton S Timonina D et al. KRAS G12C NSCLC models are sensitive to direct targeting of KRAS in combination with PI3K inhibition. *Clin Cancer Res.* (2019) 25:796–807. 10.1158/1078-0432.CCR-18-0368 30327306

[10] Riely GJ Wood DE Ettinger DS Aisner DL Akerley W Bauman JR et al. Non-Small cell lung cancer, version 4.2024, NCCN clinical practice guidelines in oncology. *J Natl Compr Canc Netw.* (2024) 22:249–74. 10.6004/jnccn.2204.0023 38754467

[11] Skoulidis F Li BT Dy GK Price TJ Falchook GS Wolf J et al. Sotorasib for lung cancers with KRAS p.G12C mutation. *N Engl J Med.* (2021) 384:2371–81. 10.1056/NEJMoa2103695 34096690 PMC9116274

[12] Jänne PA Riely GJ Gadgeel SM Heist RS Ou SI Pacheco JM et al. Adagrasib in non-small-cell lung cancer harboring a KRAS(G12C) mutation. *N Engl J Med.* (2022) 387:120–31. 10.1056/NEJMoa2204619 35658005

[13] De Langen AJ Johnson ML Mazieres J Dingemans AC Mountzios G Pless M et al. Sotorasib versus docetaxel for previously treated non-small-cell lung cancer with KRAS(G12C) mutation: a randomised, open-label, phase 3 trial. *Lancet.* (2023) 401:733–46. 10.1016/S0140-6736(23)00221-0 36764316

[14] Thota R Johnson DB Sosman JA. Trametinib in the treatment of melanoma. *Expert Opin Biol Ther.* (2015) 15:735–47. 10.1517/14712598.2015.1026323 25812921 PMC6684215

[15] Richtig E Nguyen VA Koelblinger P Wolf I Kehrer H Saxinger W et al. Dabrafenib plus trametinib in unselected advanced Braf V600-mut melanoma: a non-interventional, multicenter, prospective trial. *Melanoma Res.* (2024) 34:142–51. 10.1097/CMR.0000000000000948 38092013 PMC10906199

[16] Yan C Luo W Yang J Yang J Chen SC Bergdorf K et al. RAS/MEK/PI3K pathway inhibition augments response to CD40 agonism by targeting CD11B(+) Bregs thereby overcoming melanoma PD1-resistance. *Nat Commun.* (2026) 17:162. 10.1038/s41467-025-67315-1 41526341 PMC12796310

[17] Gadgeel SM Miao J Riess JW Moon J Mack PC Gerstner GJ et al. Phase II study of docetaxel and trametinib in patients with KRAS mutation positive recurrent non-small cell Lung cancer (NSCLC; SWOG S1507, NCT-02642042). *Clin Cancer Res.* (2023) 29:3641–9. 10.1158/1078-0432.CCR-22-3947 37233987 PMC10526968

[18] Dummer R Lebbé C Atkinson V Mandalà M Nathan PD Arance A et al. Combined PD-1, BRAF and MEK inhibition in advanced BRAF-mutant melanoma: safety run-in and biomarker cohorts of COMBI-I. *Nat Med.* (2020) 26:1557–63. 10.1038/s41591-020-1082-2 33020648

